# Optimizing protein design through uncertainty-weighted steering of protein language models

**DOI:** 10.1093/bioinformatics/btag436

**Published:** 2026-08-21

**Authors:** Alif Bin Abdul Qayyum, Yingtong Zhou, Xiaoning Qian, Byung-Jun Yoon

**Affiliations:** Department of Electrical and Computer Engineering, Texas A&M University, College Station, TX 77843, United States; Department of Electrical and Computer Engineering, Texas A&M University, College Station, TX 77843, United States; Department of Electrical and Computer Engineering, Texas A&M University, College Station, TX 77843, United States; Department of Computer Science and Engineering, Texas A&M University, College Station, TX 77843, United States; Computing and Data Sciences, Brookhaven National Laboratory, Upton, NY 11973, United States; Department of Electrical and Computer Engineering, Texas A&M University, College Station, TX 77843, United States; Computing and Data Sciences, Brookhaven National Laboratory, Upton, NY 11973, United States

## Abstract

**Motivation:**

Protein Language Models (PLMs) have revolutionized protein engineering by capturing the evolutionary constraints inherent in natural protein sequences. However, precisely steering these models to engineer novel proteins with targeted functionalities remains challenging due to the inherent difficulty in modifying their latent representations considering the design objectives. Recently, activation steering of PLMs has emerged as a potent, training-free intervention for directing PLM outputs. However, the requirement for high-quality labeled datasets limits its application. In data-scarce or out-of-distribution (OOD) regimes, researchers must rely on surrogate models for label prediction; however, deterministic surrogates fail to account for the underlying uncertainty, often yielding steering vectors that result in suboptimal protein design.

**Results:**

To address this, we propose PROSOUNDS (PROtein Sequence Optimization through UNcertainty-weighteD Steering), a PLM-based protein design framework that integrates uncertainty quantification into the activation steering logic. By weighting the steering activation calculation process based on uncertainty estimates of the surrogate predictions, PROSOUNDS enables robust protein optimization through precise mutational design even in the absence of ground-truth labels. Comprehensive performance evaluation reveals that PROSOUNDS consistently outperforms deterministic alternatives across three different protein property optimization tasks.

**Availability and Implementation:**

The datasets and implementation code for PROSOUNDS are available at https://github.com/TeresaZhouTamu/PRO-SOUNDS.

## 1 Introduction

The emergence of Protein Language Models (PLMs) has fundamentally shifted the paradigm of protein engineering from manual design to generative modeling (Rives *et al*. 2021, Elnaggar *et al*. 2022, Ferruz *et al.* 2022, Madani *et al*. 2023). By leveraging Transformer architectures trained on evolutionary-scale sequence data (ESM Team 2024, Hayes *et al*. 2025), PLMs have demonstrated an internal grasp of protein grammar, semantics, and fitness landscapes (Meier *et al*. 2021, Rao *et al*. 2021, Hie *et al*. 2022). However, a persistent challenge remains in protein engineering: controllable generation (Stanton *et al*. 2022, Ruffolo and Madani 2024, Huang *et al*. 2025). While PLMs are adept at producing biologically plausible sequences, steering them toward specific, high-value functional properties, such as enhanced immunogenicity (Li *et al*. 2025) or solubility, typically requires extensive fine-tuning or the use of labeled datasets to derive steering vectors (Ferruz and Höcker 2022, Chu *et al*. 2024, Huang *et al*. 2025).

Recent breakthroughs in Activation Steering have introduced a training-free alternative for guiding PLM outputs (Huang *et al*. 2025, Zhang *et al*. 2026). Activation Steering based Protein Optimization (ASPO) (Huang *et al*. 2025) optimizes proteins at inference time by calculating a steering vector from the mean difference between activations of high-fitness and low-fitness sequences and adding this vector to the model’s hidden states to shift the generation toward desired functional properties. While effective, this supervised approach (Huang *et al*. 2025) is inherently limited by the labeled data bottleneck: the vast majority of known protein sequences lack the precise functional annotations required to define a reliable steering direction (Radivojac *et al*. 2013).

In most practical protein engineering tasks, the number of sequences with experimentally verified properties is too small to define a robust steering direction in a high-dimensional latent design space (Yang *et al.* 2019, Wittmann *et al*. 2021). To bridge this gap, a surrogate model as a proxy for the fitness landscape, trained on the limited labeled data, is required to learn the mapping between a PLM’s internal representations and functional properties (Bae *et al*. 2025, Li *et al*. 2025). Utilizing predicted labels, provided from this trained surrogate, we can derive a steering vector that reflects the underlying biological gradients of the entire sequence space rather than just a few sparse data points.

However, deterministic surrogates are prone to overconfidence, especially when extrapolating into the dark matter of the protein space, protein sequences that are functionally distant from the training set (Greenman *et al*. 2025, Qayyum *et al*. 2026a, 2026b). Deploying probabilistic surrogate models, capable of providing not only label predictions, but also corresponding uncertainty estimates; are crucial for such out-of-distribution (OOD) scenarios (Gal and Ghahramani 2016, Hendrycks and Gimpel 2017, Li *et al*. 2022, Kim *et al*. 2025). By incorporating the surrogate model’s uncertainty into the steering vector calculation, we can weight the contribution of each sequence based on its reliability. This ensures that the resulting steering vector is guided by high-confidence, biologically grounded predictions; effectively filtering out the noise and preventing the steering process from drifting into non-functional or bio-physically unstable regions of the latent space (Kendall *et al*. 2018, Filos *et al.* 2020, Eysenbach *et al*. 2021).

We propose *PROSOUNDS* (*PRO*tein *S*equence *O*ptimization through *UN*certainty-weighte*D S*teering), an uncertainty-aware framework that modulates the internal activations of PLMs by integrating uncertainty estimates from probabilistic property predicting surrogate models, into the activation steering process to guide property-specific sequence optimization.

## 2 Methodology

We propose a novel *Activation Steering for PLMs* in which the PLMs’ internal representations are modulated during inference. Our proposed *PROSOUNDS* approach integrates uncertainty estimates into activations of positive and negative samples to promote the generation of sequences satisfying a desired functional property.

### 2.1 Problem formulation

We assume access to two distinct data distributions: (i) a labeled source domain $D_{\text{train}}$, and (ii) an unlabeled target domain $D_{\text{test}}$.

The source domain is defined as


(1) $$D_{\text{train}}={\left\{(x_{i},y_{i})\right\}}_{i=1}^{N},\;(x,y)∼P_{\text{source}}(x,y),$$

Where *x* denotes experimentally verified protein sequences, and *y* denotes their corresponding property labels. This dataset serves as the ground-truth anchor for the desired property, but it may be limited in size and biased toward well-studied protein families that are intrinsically different from the user’s area of interest.

The target domain is defined as


(2) $$D_{\text{test}}={\left\{x_{j}^{\prime }\right\}}_{j=1}^{M},\;x^{\prime }∼P_{\text{target}}(x),$$

An unlabeled dataset representing the unknown/unexplored protein manifold, regions of non-characterized sequence space, yet relevant to the user’s design objectives.

A critical barrier in this setup is the *distributional shift* between the source and target domains, i.e.


(3) $$P_{\text{source}}(x,y)\neq P_{\text{target}}(x,y).$$

Given $x\in D_{\mathrm{test}}$, the objective is to mutate *x* into $x^{\prime }$ to optimize its property value $y(x^{\prime })$ relative to the baseline sequence y(x).

### 2.2 PROSOUNDS

To guide protein generation toward desired properties, ASPO (Huang *et al*. 2025) employs activation steering by modifying the $l^{th}$ layer activations of the protein language model, $h_{l}\in R^{L\times d}$ (where *L* is the sequence length and *d* is the hidden dimension of the model), with a scalar steering strength, β, as follows:


(4) $${\tilde{h}}_{l}=\;h_{l}+\beta v_{l}$$

To design the steering vector $v_{l}\in R^{d}$, ASPO (Huang *et al*. 2025) proposes to utilize positive and negative samples from the source domain $D_{\text{train}}$, and extract the hidden state embeddings of those sequences in the following manner to compute $v_{l}$:


(5) $$v_{l}=\frac{1}{\left|P\right|}\sum_{x_{p}\in P}h_{l}^{\mathrm{avg}}(x_{p})-\frac{1}{\left|N\right|}\sum_{x_{n}\in N}h_{l}^{\mathrm{avg}}(x_{n})$$

Here, P and N denote the positive and negative set of samples in $D_{\text{train}}$ respectively, and $h_{l}^{\mathrm{avg}}\in R^{d}$ denotes the average activations across all tokens in the sequence.

Due to the distributional shift in $D_{\text{test}}$ from $D_{\text{train}}$, we can not directly use protein sequences sampled from the source distribution $P_{\text{source}}$, as employed in ASPO (Huang *et al*. 2025). Rather, we need to design a surrogate model that can predict the desired property of interest for protein sequences sampled from $P_{\text{target}}$.

Standard surrogate models trained solely on $D_{\text{train}}$ often suffer from Out-of-Distribution (OOD) hallucination when applied to $D_{\text{test}}$, predicting high functional scores with unjustified confidence for sequences that may be non-functional or bio-physically unstable.

Following the training-free approach of ASPO (Huang *et al*. 2025), we employ the PROSOUNDS framework which maintains a training-free generation process while mitigating OOD hallucinations.

PROSOUNDS proposes a hybrid framework to calculate $v_{\text{hybrid}}^{\left(l\right)}$, instead of a simplistic $v_{l}$ measured only through samples from $D_{\text{train}}$:


(6) $$v_{\text{hybrid}}^{\left(l\right)}=\alpha_{l}v_{\text{property}}^{\left(l\right)}+(1-\alpha_{l})v_{\text{test}}^{\left(l\right)}$$

PROSOUNDS leverages (i) probabilistic surrogate model to infer property labels for $D_{\text{test}}$ along with epistemic uncertainty (Hüllermeier and Waegeman 2021) estimates of these predictions that we use to weight the activation steering vectors and measure $v_{\text{test}}^{\left(l\right)}$, and (ii) dual-domain hybrid steering approach to utilize samples from not only the target domain with predicted labels, but also samples from source domain with experimentally validated labels, through measuring $v_{\text{property}}^{\left(l\right)}$ and then the final hybrid steering vector $v_{\text{hybrid}}^{\left(l\right)}$.

Our approach comprises two key components: (i) Uncertainty Weighted Activation Steering, and (ii) Dual-Domain Hybrid Steering.

#### 2.2.1 Uncertainty weighted activation steering

For each sequence $x^{\prime }\in D_{\text{test}}$, the probabilistic surrogate we employ produces a point estimate for the property, $p(y|x^{\prime })$ and the corresponding epistemic uncertainty $u(x^{\prime })$. We leverage an uncertainty-aware weighting schemes to evaluate $v_{\text{test}}^{\left(l\right)}$.

We first partition $D_{\text{test}}$ into pseudo-positive and pseudo-negative subsets based on surrogate predictions. As predicted class distributions are often imbalanced, we avoid fixed probability thresholds. Instead, within each predicted class, samples are ranked by their epistemic uncertainty $u_{i}$, and we retain the top-*N* most certain instances from each group, where *N* is bounded by the size of the minority class. This ensures balanced pseudo-positive set $P_{\text{test}}$ and pseudo-negative set $N_{\text{test}}$, preventing majority-class dominance in the steering computation.

The target steering vector at layer *l* is defined as the difference between the uncertainty-weighted centroids of these subsets:


(7) $$v_{\text{test}}^{\left(l\right)}=\frac{\sum_{x_{i}\in P_{\text{test}}}w_{i}^{\text{pos}}h_{l}^{\mathrm{avg}}(x_{i})}{\sum_{x_{i}\in P_{\text{test}}}w_{i}^{\text{pos}}}-\frac{\sum_{x_{j}\in N_{\text{test}}}w_{j}^{\text{neg}}h_{l}^{\mathrm{avg}}(x_{j})}{\sum_{x_{j}\in N_{\text{test}}}w_{j}^{\text{neg}}}$$

Where $h_{l}^{\mathrm{avg}}(x)$ denotes the token-averaged hidden representation of sequence *x* at layer l∈{1,…,L}.

To design $w_{i}^{\text{pos}}$ and $w_{j}^{\text{neg}}$, we integrate quantified epistemic uncertainty (EU) (Hüllermeier and Waegeman 2021) into the sample weighting process under two complementary statistical objectives: (i) variance minimization and (ii) risk-averse selection.

*Precision-based Reliability Weighting*: To minimize the variance of the estimated steering direction, we assume that surrogate prediction errors are normally distributed with variance proportional to epistemic uncertainty.


(8) $$\text{Var}({\hat{y}}_{i})\propto u_{i}^{2}$$

Under this assumption, the Minimum Variance Unbiased Estimator is obtained by weighting each sample inversely proportional to its variance. We therefore define a *Precision-based Reliability Weight*


(9) $$w_{i}=\frac{1}{\max{\left(u_{i},\epsilon \right)}^{2}},$$

Where $u_{i}$ denotes the epistemic uncertainty associated with sequence $x_{i}$, and ϵ is a Winsorizing threshold defined as the $10^{th}$ percentile of the uncertainty distribution. This truncation prevents samples with extremely small uncertainty estimates, often caused by model overconfidence, from disproportionately dominating the steering vector estimation.

*Risk-averse Weighting*: Inverse-variance weighting alone does not explicitly guard against false positives arising from distributional shift. To mitigate this risk, we adopt a conservative selection principle inspired by the Lower Confidence Bound (LCB) formulation. Rather than relying on the expected surrogate prediction probability ${\hat{p}}_{i}$, we penalize it by its associated epistemic uncertainty:


(10) $$w_{i}^{\text{pos}}={\left(\max({\hat{p}}_{i}-\lambda u_{i},0)\right)}^{\gamma }.$$

(11) $$w_{i}^{\text{neg}}={\left(\max(1-{\hat{p}}_{i}-\lambda u_{i},0)\right)}^{\gamma }.$$

Here, λ=1.0 controls the degree of risk aversion, and γ=2.0 sharpens the contrast between reliable and unreliable samples. This *Risk-averse Weight* formulation prevents the steering vector from being contaminated by out-of-distribution samples exhibiting large predictive variance.

#### 2.2.2 Dual-domain hybrid steering

Relying on a single data domain introduces a fundamental trade-off: the source domain $D_{\text{train}}$ provides reliability through ground-truth labels but may lack distributional relevance, whereas the target domain $D_{\text{test}}$ offers domain specificity but suffers from surrogate-induced label noise.

To mitigate the risks associated with unverified target-domain steering, we additionally compute a source-domain steering vector $v_{\text{train}}^{\left(l\right)}$ using the ground-truth labels from $D_{\text{train}}$ following Equation 5. Although the source distribution may differ from the user’s specific target cluster, it encodes experimentally verified biophysical constraints that are broadly invariant across domains.

Simply averaging $v_{\text{train}}^{\left(l\right)}$ and $v_{\text{test}}^{\left(l\right)}$ is suboptimal, as the source vector may encode domain-specific biases that could shift generation away from the target distribution. To integrate these signals while preventing distributional drift, we introduce a *Gated Hybrid Fusion* mechanism, to disentangle the functional signal in the source domain from its distributional bias.

Let the cluster axis be defined as the vector connecting the global centroids of the target and source domains:


(12) $$v_{\text{cluster}}^{\left(l\right)}=\mu_{\text{test}}^{\left(l\right)}-\mu_{\text{train}}^{\left(l\right)},$$

Where $\mu_{\text{train}}^{\left(l\right)}$ and $\mu_{\text{test}}^{\left(l\right)}$ denote the layer-*l* global centroids of $D_{\text{train}}$ and $D_{\text{test}}$, respectively. To isolate the functional component of the source steering direction, we project $v_{\text{train}}^{\left(l\right)}$ onto the null space of $v_{\text{cluster}}^{\left(l\right)}$ using Gram–Schmidt orthogonalization:


(13) $$v_{\text{property}}^{\left(l\right)}=v_{\text{train}}^{\left(l\right)}-{\text{proj}}_{v_{\text{cluster}}^{\left(l\right)}}\left(v_{\text{train}}^{\left(l\right)}\right),$$

(14) $${\text{proj}}_{a}(b)=\frac{\langle b,a\rangle }{\|a{\|}^{2}}a.$$

The resulting vector $v_{\text{property}}^{\left(l\right)}$ preserves the experimentally verified functional direction while removing the component aligned with domain shift.

As summarized in Algorithm 1, we fuse $v_{\text{property}}^{\left(l\right)}$ with $v_{\text{test}}^{\left(l\right)}$ via a layer-wise mixing coefficient $\alpha_{l}$ (mentioned in Equation 6). PLMs tend to focus on high-level functional abstractions in deeper layers (Vig *et al*. 2021), and to comply with this tendency of PLMs, $\alpha_{l}$ follows a linear decay schedule, decreasing from 1.0 in early layers to 0.0 in deeper layers to ensure the dominance of $v_{\text{test}}^{\left(l\right)}$ over $v_{\text{hybrid}}^{\left(l\right)}$ for higher *l*s. To safeguard against catastrophic steering when noisy target directions conflict with experimentally validated biological constraints, we introduce a conflict-aware gating mechanism based on cosine similarity. This gated mechanism ensures biological plausibility by defaulting to safe, experimentally verified source data during conflicts, while still allowing the model to adapt to target-specific nuances.

For sequence optimization, we follow the ASPO framework (Huang *et al*. 2025) to select an injection layer $l^{*}$ with maximal linear separability between pseudo-positive and pseudo-negative samples. In each optimization round, tokens that are most misaligned with the hybrid vector are masked and regenerated under activation steering (Equation 4), iteratively optimizing the sequence toward the desired functional property (Algorithm 1).

Algorithm 1 Hybrid Dual-Domain Activation Steering

**Require:** Source vectors $v_{\text{property}}^{\left(l\right)}$, target vectors $v_{\text{test}}^{\left(l\right)}$, sequence $x_{0}$, rounds *R*, mutation budget *T*

**Ensure:** Optimized sequence $x_{R}$

1: **for**  l=1 to *L* **do** 

2:   $\text{sim}\leftarrow \text{CosineSimilarity}(v_{\text{property}}^{\left(l\right)},v_{\text{test}}^{\left(l\right)})$

3:   $\alpha_{l}\leftarrow 1.0$ if sim<τ, else LinearDecay(l)

4:   $v_{\text{hybrid}}^{\left(l\right)}\leftarrow \alpha_{l}v_{\text{property}}^{\left(l\right)}+(1-\alpha_{l})v_{\text{test}}^{\left(l\right)}$

5: **end for** 

6: **for**  r=1 to *R* **do** 

7:   **for all** token *j* in $x_{r-1}$  **do** 

8:     $s_{j}\leftarrow \text{CosineSimilarity}(h_{j}^{\left(l^{*}\right)}(x_{r-1}),v_{\text{hybrid}}^{\left(l^{*}\right)})$

9:   **end for** 

10:   Select *T* positions with smallest $s_{j}$ and regenerate using $v_{\text{hybrid}}^{\left(l^{*}\right)}$

11:   $x_{r}\leftarrow$ updated sequence

12: **end for** 

    **return**  $x_{R}$

## 3 Experiments

We empirically evaluate PROSOUNDS across multiple functional optimization tasks. We consider three biologically relevant objectives: optimization of (i) *immunogenicity*, (ii) *solubility*, and (iii) *toxicity*.

### 3.1 Experimental setup

*Dataset*: Experiments are conducted on three datasets: ImmunoDB dataset (Li *et al*. 2025) for immunogenicity, the DeepSol dataset (Khurana *et al*. 2018) for solubility, and ToxDL2.0 dataset (Zhu *et al*. 2025) for toxicity. Target sequences are treated as unlabeled during steering vector construction to reflect realistic deployment constraints. Optimization outcomes are evaluated using independent proxy predictors: the fine-tuned VenusVaccine (VV) model (Li *et al*. 2025) for immunogenicity and toxicity, and the fitness predictor from (Huang *et al*. 2025) for solubility.

*Data Splitting*: To enforce substantial distributional shift and avoid trivial generalization, we construct $D_{\text{train}}$ and $D_{\text{test}}$ using clustering-based partitioning rather than random splits. For the immunogenicity task, the largest BLOSUM62-induced cluster is designated as the source domain, while the most phylogenetically distant cluster serves as the out-of-distribution (OOD) target domain. For solubility and toxicity, the most geometrically distant *K*-means clusters of the 3-gram TF-IDF features are selected as target domains to maximize distributional separation.

### 3.2 Probabilistic surrogate models

To quantify epistemic uncertainty for weighted steering, we instantiate *PRO-SOUNDS* with three probabilistic surrogate backbones, all sharing the VenusVaccine (Li *et al*. 2025) architecture as the base encoder.

1. *MC Dropout* estimates epistemic uncertainty via multiple stochastic forward passes with dropout enabled at inference, using predictive entropy over the resulting ensemble (Gal and Ghahramani 2016).
2. *Evidential Deep Learning (EDL)* replaces the output layer with a Dirichlet parameterization, deriving uncertainty from the total accumulated evidence (Sensoy *et al*. 2018).
3. *Flexible EDL (FEDL)* extends EDL with transport-based regularization to mitigate overconfidence in low-support regions, yielding improved uncertainty calibration (Yoon and Kim 2025).

### 3.3 PROSOUNDS variants

We adopt ASPO (Huang *et al*. 2025) as the baseline for this work. We evaluate PROSOUNDS against this baseline, instantiated with the three different probabilistic backbones. For each backbone, we examine four distinct steering vector formulations:

- Precision Weighted Target Vector: This is constructed solely from pseudo-labeled target samples and weighted by the inverse predicted epistemic uncertainty following Equation 8. For this variant, we set $\alpha_{l}$ to 0 for all layers (see Algorithm 1).
- Risk Averse Target Vector: This applies strict penalization to filter high-uncertainty samples following Equations 10 and 11. Similar to the previous one, we set $\alpha_{l}$ to 0 for all layers (see Algorithm 1).
- Hybrid Precision Fusion: A gated combination of precision-weighted target vectors (following Equation 8) and source-domain steering vectors, following Equation 6.
- Hybrid Risk Averse Fusion: A gated combination of risk-averse target vectors (following Equations 10 and 11) and source-domain steering vectors, following Equation 6.

### 3.4 Hyperparameters

Across all experiments, we fix the combined size of the positive and negative sets to approximately 350 samples and β=1.0, with optimization limited to R=4 rounds and T=2 mutations per round. The conservative limits on *R* and *T* act as regularization to prevent sequence degeneracy. Although larger mutation budgets can inflate surrogate fitness, they tend to produce repetitive, biologically implausible motifs. These constraints reduce drift from the wild-type manifold and promote stable convergence toward structurally realistic, functional sequences. All steering experiments are conducted on the ESMC 600M version of the ESMC model (ESM Team 2024) with the hidden dimension d=1152.

## 4 Results and analysi

### 4.1 Comparative evaluations

**Figure 1:**
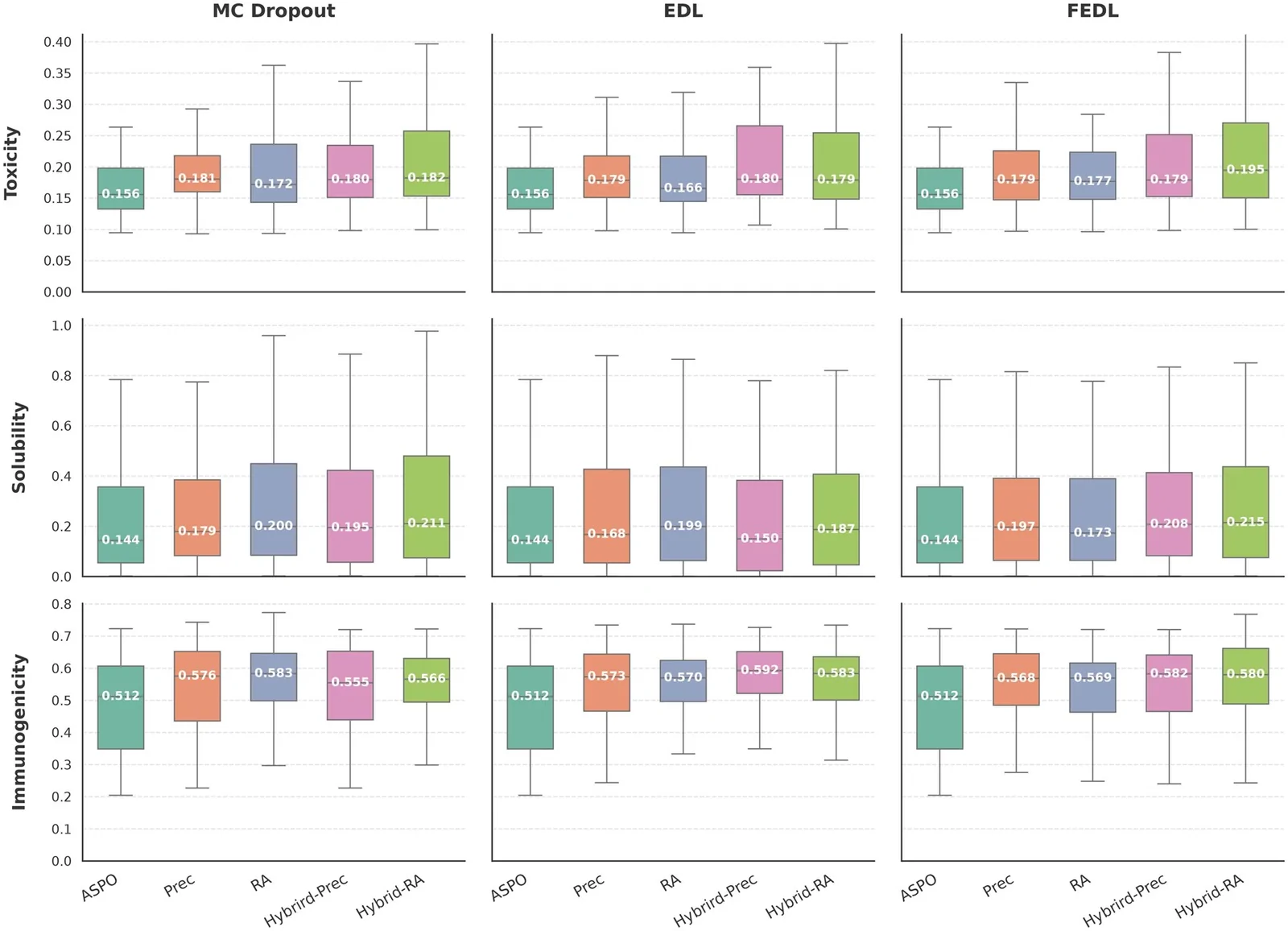
Optimization performance across surrogate backbones and steering strategies. The 3×3 grid shows the distributions of optimization outcomes for three protein property tasks (Immunogenicity, Solubility, and Toxicity; rows) across three probabilistic surrogate backbones (MC Dropout, EDL, and FEDL; columns). Steering strategies include the ASPO baseline and four uncertainty-guided variants of *PRO-SOUNDS: Precision Weighted Target Vector* (Prec), *Risk Averse Target Vector* (RA), *Hybrid Precision Fusion* (Hybrid-Prec), and *Hybrid Risk Averse Fusion* (Hybrid-RA). Across tasks and surrogate models, hybrid variants consistently achieve higher and more stable fitness improvements, particularly under out-of-distribution optimization regimes.

Across immunogenicity, solubility, and toxicity optimization tasks shown in Fig. 1, all PROSOUNDS variants consistently outperform the baseline ASPO method, with the largest gains achieved through gated hybrid fusion.

As shown in Fig. 1, PROSOUNDS variants consistently outperform the baseline ASPO method, with the largest gains achieved through gated hybrid fusion. Statistical testing confirms these fitness improvements are highly significant across all variants for immunogenicity, and specifically for hybrid formulations in the challenging toxicity and solubility domains (p<0.05; one-sided Mann-Whitney *U* test, see Table S1, available as supplementary data at *Bioinformatics* online).

In immunogenicity optimization, hybrid risk-averse configurations deliver the most robust performance, with the EDL variant attaining the highest median fitness (0.5835) and the FEDL hybrid dominating the upper tail (75th percentile: 0.6617).

For solubility optimization under strict structural splits, uncertainty-aware reweighting proves critical: the EDL risk-averse baseline improves the median to 0.1993, while the FEDL hybrid risk-averse strategy achieves the strongest overall performance (median 0.2148, 75th percentile 0.4373, p=0.0435).

In toxicity optimization, while precision weighting with MC Dropout increases median fitness (0.1806), the FEDL hybrid risk-averse variant provides the best balance between exploration and structural constraint (median 0.1948, 75th percentile 0.2703, p=0.0078).

Collectively, these results demonstrate that integrating risk-averse target steering with source-domain anchoring enables PROSOUNDS to reliably traverse high-fitness regions that remain inaccessible to standard steering approaches.

### 4.2 Epistemic uncertainty as a high-fidelity signal

**Figure 2:**
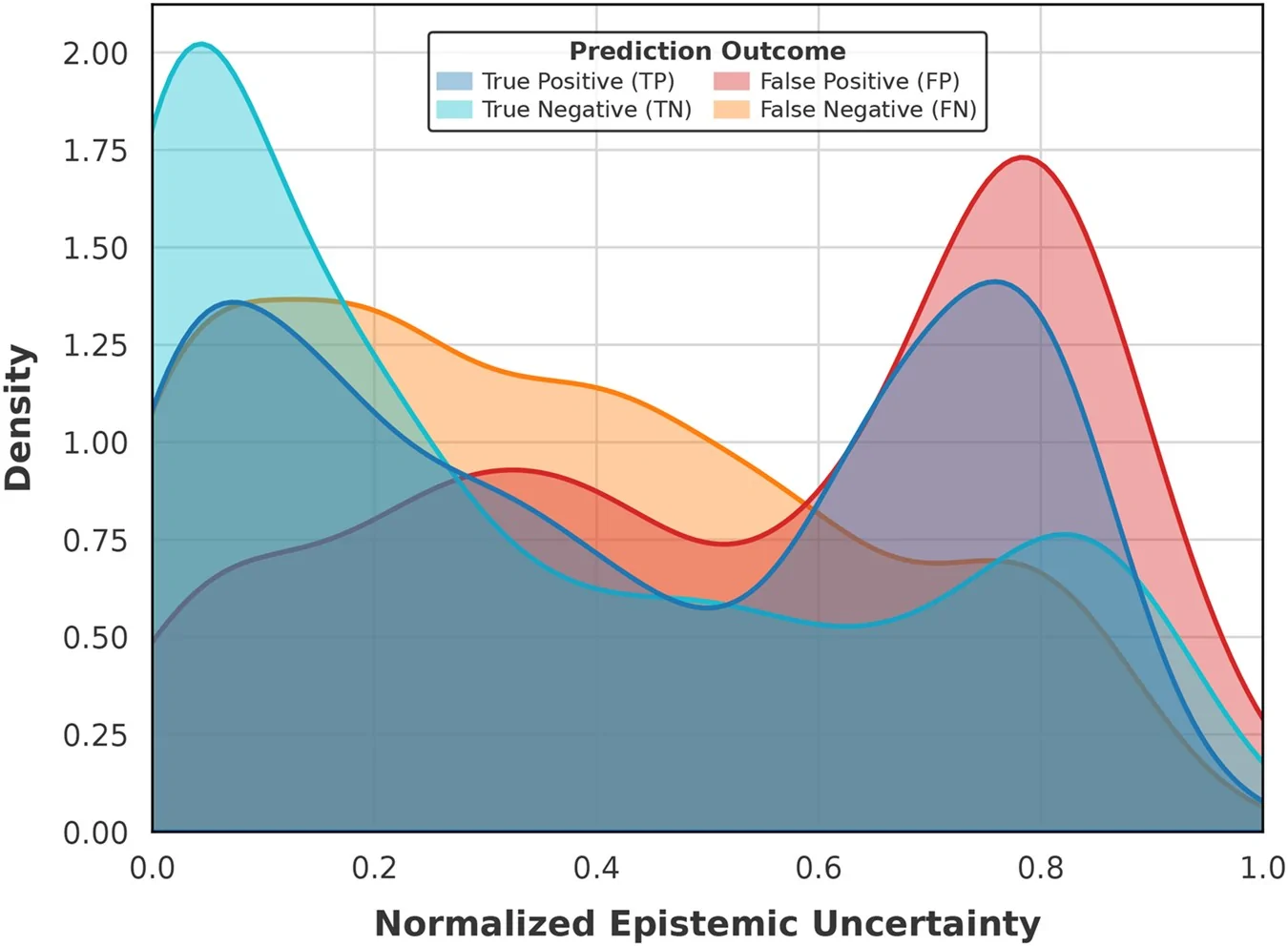
Predictive uncertainty across outcome categories. Density distributions of normalized epistemic uncertainty across four prediction outcomes. Correct predictions (TP, TN) are concentrated at low uncertainty, while incorrect predictions (FP, FN) exhibit elevated uncertainty. Only the TP case displays two peaks, one at low and another at high uncertainty region, deviating from the overall trend.

Our core motivation for utilizing epistemic uncertainty (EU) as a reweighting mechanism is to systematically penalize incorrect surrogate predictions during optimization. As demonstrated in Fig. 2, EU serves as a high-fidelity indicator of prediction error. The overall trend reveals that accurate predictions, both True Positives (TP) and True Negatives (TN), predominantly concentrate in the low-EU region; whereas incorrect predictions, False Positives (FP) and False Negatives (FN), maintain substantial density at higher EU values.

Notable exceptions to this trend are observed in the TP and FN categories. The TP distribution shows a secondary density mass in the high-uncertainty regime, reflecting instances where correct predictions in extreme out-of-distribution spaces are inherently accompanied by elevated model uncertainty, while a subset of FNs conversely extends into the lower-uncertainty space.

Downweighting high-EU samples introduces an unavoidable side effect: it suppresses some uncertain true positives (TPs) while upweighting low-EU false negatives (FNs). Although both cases represent missed optimization opportunities relative to an ideal reweighting scheme, this cost is justified by the need to eliminate risky false positives (FPs). While the misweighting of TPs and FNs only delays the optimization process, incorporating high-EU FPs actively poisons the generative trajectory. By accepting this trade-off, PROSOUNDS successfully filters out the most harmful gradients, ensuring robust out-of-distribution generalization (Fig. 1).

### 4.3 A special case: geometric constraints and adaptation of hybrid fusion

**Figure 3:**
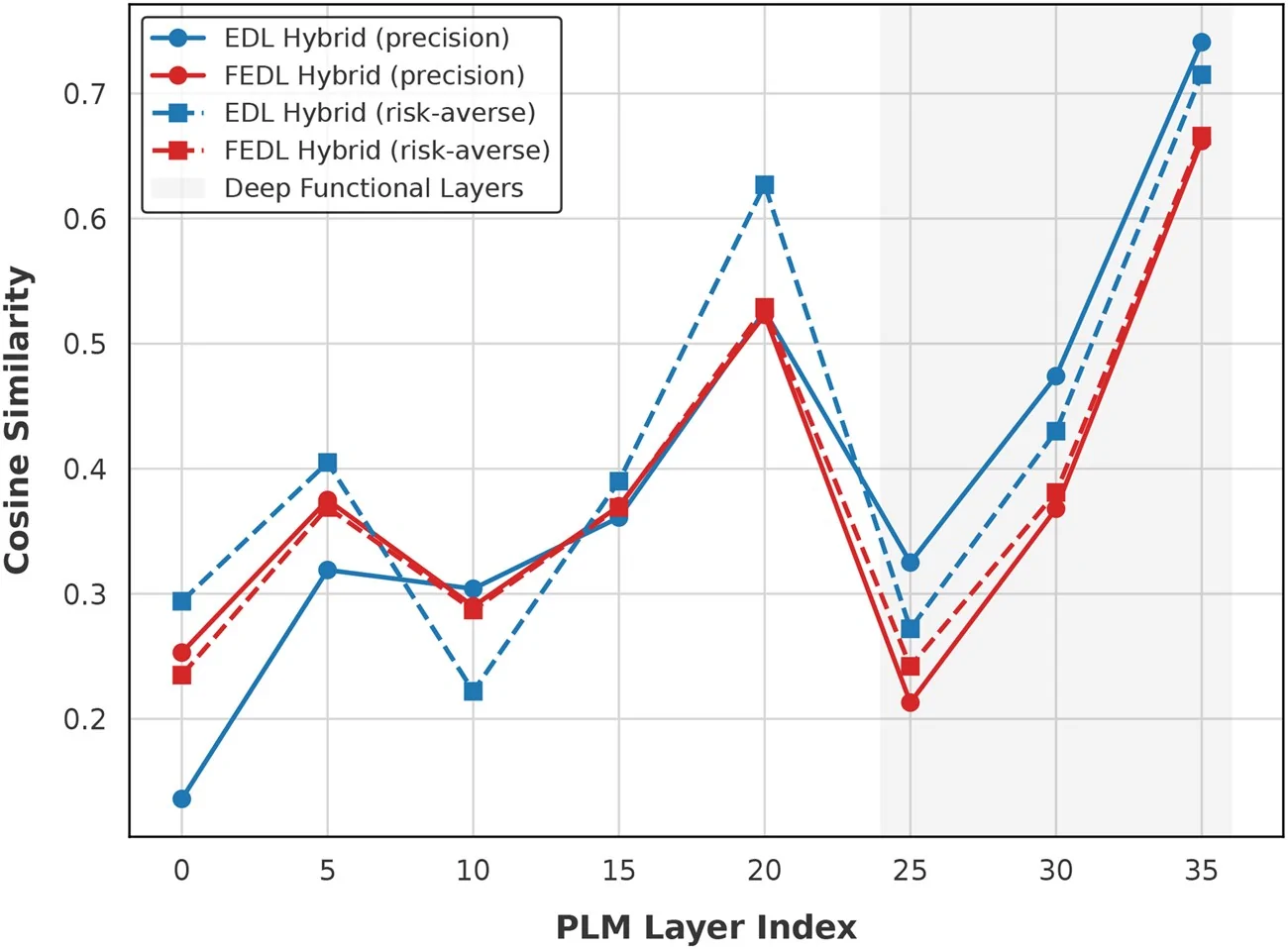
Layer-wise cosine similarity of steering vectors during optimization. Cosine similarity between source and target vectors across the 36 layers of the ESMC model during solubility optimization. The shaded region denotes deep, semantically rich layers (Layers 24–36). FEDL-guided hybrid variants show lower deep-layer similarity than EDL-guided variants, indicating reduced feature entanglement and more orthogonal trajectories into out-of-distribution space.

While EU-driven reweighting establishes a robust foundational trajectory, the gains from gated hybrid fusion can be constrained by geometric alignment conditions within the PLM. We specifically focus our layer-wise analysis on the EDL surrogate for solubility optimization (Fig. 3), as it represents a unique outlier across our experiments. Unlike the immunogenicity and toxicity tasks—where hybrid fusion consistently enhances fitness—EDL hybrid steering for solubility paradoxically underperforms in Fig. 1 and Table S1, available as supplementary data at *Bioinformatics* online. In these rare cases where hybrid fusion fails to improve fitness, we observe high cosine similarity between source and target steering vectors in the deeper, semantically enriched PLM layers (Vig *et al*. 2021). These layers encode complex functional abstractions, and elevated similarity indicates feature entanglement rather than complementary information.

Crucially, the layer-wise linear decay weight $\alpha_{l}$ amplifies this vulnerability. Because $\alpha_{l}$ is designed to decay toward zero in deeper layers to prioritize target-domain exploration, it heavily penalizes the source signal. In highly entangled scenarios, the target vector largely mirrors the source representation rather than introducing an orthogonal direction into the unexplored OOD space. Consequently, the linear decay mechanism discards the stable, known source anchor without gaining any meaningful exploratory guidance from the target vector, fundamentally limiting the potential benefits of multi-domain fusion and explaining the isolated performance drop observed in this specific dataset split.

## 5 Discussion

We consistently observe a performance advantage when the risk-averse strategy is combined with the gated hybrid mechanism (Fig. 1). Under isolated target steering, precision-based and risk-averse formulations show comparable performance, reflecting a trade-off between maintaining geometric diversity and suppressing out-of-distribution noise. However, in the hybrid setting, risk-averse configurations dominate by enforcing a hard threshold that zeroes out highly uncertain samples, eliminating residual noise and yielding a compact, low-variance target vector. Within the hybrid framework, this strict filtering integrates optimally with the structurally stable source-domain anchor, producing a highly specific and reliable functional trajectory that enables superior optimization performance.

## 6 Conclusion

In this work, we introduced PROSOUNDS, an uncertainty-aware protein design framework that addresses the critical challenge of steering Protein Language Models in data-scarce and out-of-distribution (OOD) scenarios for property optimization. Our findings demonstrate that integrating rigorous uncertainty quantification into the activation steering logic provides a robust mechanism for protein optimization. We introduced multiple PROSOUNDS variants and benchmarked their performance across three distinct protein property optimization tasks. Extensive experimental results demonstrate that uncertainty-aware steering of PLMs consistently surpasses deterministic baselines, particularly under distributional shift, which is fairly common in real-world design scenarios.

PROSOUNDS advances our understanding of how learned functional constraints generalize to out-of-distribution sequences. By coupling hybrid source-target fusion with uncertainty gating, our method reveals that successful domain adaptation depends on isolating structural representations characterized by low epistemic uncertainty. These low-EU features encapsulate robust, universally transferable biophysical rules. By anchoring the generative trajectory to these stable functional signals, PROSOUNDS successfully bridges the gap between domains, extending reliable sequence representations into unexplored regions while effectively filtering out non-transferable, domain-specific noise.

By effectively weighting activations according to the epistemic uncertainty, our approach mitigates the risks associated with sub-optimal label estimation in OOD settings. This work lays the foundation for more reliable, uncertainty-aware protein engineering, providing a scalable path toward robust mutational design in the absence of dense, ground-truth experimental labels.

## Supplementary Material

btag436_Supplementary_Data

## Data Availability

The datasets and implementation code for PROSOUNDS are available at https://github.com/TeresaZhouTamu/PRO-SOUNDS.
