# Supplementary material for: Optimizing protein design through uncertainty-weighted steering of protein language models
Source: Bioinformatics. 2026 Aug 21;42(Suppl 2):btag436. doi: 10.1093/bioinformatics/btag436 (PMC13501300; doi:10.1093/bioinformatics/btag436)
Supplement: Supplementary file 1 [file btag436_supplementary_data.pdf]

Supplementary Materials

Alif Bin Abdul Qayyum, Yingtongz Zhou, Xiaoning Qian
and Byung-Jun Yoon

1 Related Works

Protein Language Models (PLM) have demonstrated significant generative potential by learning the underlying distribution of the protein sequence space through masked [3, 4, 7, 25] or auto-regressive [6, 19] language modeling objectives. Beyond their primary generative capacity, these models excel in tasks such as predicting mutation effects without supervision [20] and simulating evolutionary paths [9]. Their success in structure-aware design stems from a latent encoding of biophysical properties that similarly drives top-tier performance in fitness forecasting [10, 15] and structural inference [16]. While PLMs have been adapted for multi-task settings [18, 24] and conditional protein generation [5, 18, 19], the reliable steering of generated sequences is still in its infancy. In contrast to natural language processing, protein engineering demands a rigorous adherence to structural viability and evolutionary plausibility. These inherent biophysical constraints frequently diminish the efficacy of conventional Large Language Model (LLM) control strategies, such as prompting and fine-tuning, which are often insufficient for navigating the complex sequence-function landscape.

Activation Steering modulates model behavior at inference time by perturbing internal activations without retraining model weights. Activation Addition (ActAdd) computes steering vectors from contrasting prompt pairs (e.g., truthful vs. deceptive) to shift hidden states [28], a technique further stabilized by Contrastive Activation Addition (CAA) through the aggregation of hundreds of contrast pairs [23]. Subsequent refinements have utilized dataset preferences [14], affine steering [27], and bi-level optimization [2] to achieve more precise control. These methods effectively steer attributes like safety, style, and truthfulness [17, 32], supporting applications in bias mitigation [1] and adversarial robustness [29].

Protein Language Model Activation Steering has extended activation steering idea into the biological domain [11]. Activation Steering based Protein Optimization (ASPO) [11] optimizes proteins at inference time by calculating a steering vector from the mean difference between activations of high-fitness and low-fitness sequences and adding this vector to the model's hidden states to shift the generation toward desired functional properties. Inspired by the activation steering approach, Utility-Controlled Contrastive Steering (UCCS) [31] utilizes disentangled steering vectors derived from structural utility-constrained datasets to suppress pathological repetition while preserving the folding reliability of generated proteins.

While these methods demonstrate the versatility of activation steering for both functional and structural control, they typically rely on labeled dataset, or at least a surrogate model to get the labels for measuring the steering vector. However, deterministic surrogates often fail to quantify predictive uncertainty, a limitation that becomes particularly acute when generating labels for out-of-distribution (OOD) sequences [8, 12, 13, 21, 22, 26, 30]. To harness the combined benefits of predictive uncertainty estimates and inference-time activation steering, it is essential to integrate uncertainty estimates directly into the activation steering framework, ensuring that latent interventions are moderated by the model's confidence in the underlying sequence-function landscape.

2 Dataset Partitions

2.1 Domain Partitioning and Data Processing

For the immunogenicity task, the largest BLOSUM62-induced cluster is designated as the source domain, while the most phylogenetically distant cluster serves as the out-of-distribution (OOD) target domain. For solubility and toxicity, the most geometrically distant K-means clusters of the 3-gram TF-IDF features are selected as target domains to maximize distributional separation, with $K = 2$ used for solubility and $K = 7$ used for toxicity. The distinct sequence manifold separation achieved between these source and target domains across all three tasks is visually confirmed via t-SNE projections in Figure 1. Within each OOD target cluster, we randomly sample exactly 100 negative sequences as test instances for optimization. To ensure that performance improvements arose from meaningful representation adaptation rather than shifts in class priors, strict class balancing is enforced in both $\mathcal{D}_{\text{train}}$ and $\mathcal{D}_{\text{test}}$ across all tasks. The exact sequence counts for the full datasets and the resulting partitioned domains are detailed in Table 1.

Notably, to quantitatively evaluate optimization success without information leakage, the proxy ground-truth predictors are rigorously isolated from data used to construct the steering vectors for optimization. The VenusVaccine (VV)

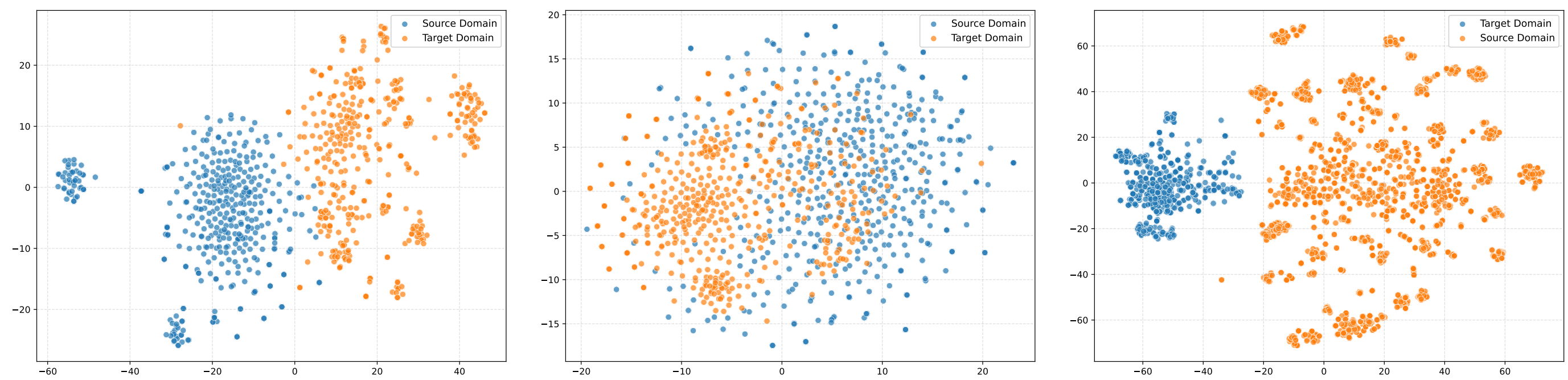

(a) (b) (c)

Figure 1: **t-SNE visualization of sequence manifold separation across three property optimization tasks.** The 2D projections illustrate the distinct geometrical separation achieved between the designated source domains and the out-of-distribution (OOD) target domains for (a) toxicity, (b) solubility, and (c) immunogenicity. The explicit isolation of distant clusters confirms the distributional shift required to rigorously evaluate OOD generalization and representation adaptation without information leakage.

Table 1: Dataset partition sizes for property optimization tasks. The table details the total number of sequences in the original datasets, alongside the specific sequence counts comprising the extracted source and out-of-distribution (OOD) target domains following sequence clustering.

**Property Task**	**Full Dataset**	**Source Domain**	**Target Domain**
Toxicity	17235	1184	406
Solubility	71421	6250	500
Immunogenicity	3964	2716	615

models [15] for immunogenicity and toxicity are trained on disjoint data partitions respectively, explicitly excluding all sequences used in $\mathcal{D}_{\text{train}}$ and $\mathcal{D}_{\text{test}}$. Similarly, the solubility fitness predictor [11] is trained solely on the remaining partition of the DeepSol data, ensuring that performance metrics reflect true out-of-distribution generalization rather than memorization.

3 Probabilistic Surrogate Variants and Uncertainty Estimation

To effectively guide the optimization trajectory and mitigate out-of-distribution (OOD) failures, we instantiate three distinct probabilistic surrogate variants. Each variant is formulated for a K-class classification setting, where $K = 2$ for our binary properties.

3.0.1 Monte Carlo (MC) Dropout

The first variant relies on standard training on $\mathcal{D}_{\text{train}}$ and Monte Carlo dropout inference on $\mathcal{D}_{\text{test}}$. Let p_t be the probability vector for the t-th sample (the output of the softmax layer), and let T be the total number of MC samples (forward passes). The expected probability is given by:

$$\bar{p} = \frac{1}{T} \sum_{t=1}^{T} p_t \tag{1}$$

Using the entropy function $H(p) = -\sum_{k=1}^{K} p_k \ln(p_k)$, the epistemic uncertainty is calculated as the entropy of the expected predictive distribution minus the expected entropy of the individual predictions:

$$U_{\text{epistemic}} = H(\bar{p}) - \frac{1}{T} \sum_{t=1}^{T} H(p_t) \tag{2}$$

3.0.2 Evidential Deep Learning (EDL)

The second variant uses training with the EDL loss on $\mathcal{D}_{\text{train}}$ and standard deterministic inference on $\mathcal{D}_{\text{test}}$. The epistemic uncertainty is calculated directly from the network logits:

$$U_{\text{epistemic}} = \frac{K}{\sum_{c=1}^{K}(e^{\text{logit}_c} + 1)} \tag{3}$$

3.0.3 Flexible Evidential Deep Learning (FEDL)

The final variant utilizes training with the Flexible Evidential Deep Learning (FEDL) loss on $\mathcal{D}_{\text{train}}$ and standard inference on $\mathcal{D}_{\text{test}}$. The trained surrogate provides the intermediate parameters α_k, γ, and p_k. The total evidence state is parameterized as:

$$\alpha_0 = \sum_{k=1}^{K} \alpha_k \tag{4}$$

$$S = \alpha_0 + \gamma \tag{5}$$

$$\tilde{\alpha}_k = \alpha_k + p_k S \tag{6}$$

Using these parameters, the epistemic uncertainty formulation is computed as:

$$U_{\text{epistemic}} = \sum_{k=1}^{K} \left[\frac{\alpha_k(1-\alpha_k)}{S+1} + \frac{2p_k(1-p_k)}{S(S+1)} \right] \tag{7}$$

Supplementary Note S1: Statistical Analysis of Fitness Enhancements

Table 2 presents a comprehensive breakdown of the statistical significance of fitness improvements achieved by various PROSOUNDS formulations compared to the ASPO baseline. To rigorously evaluate the performance gains, we conducted one-sided Mann-Whitney U tests ($N = 100$, $\alpha = 0.05$) on predicted probabilities from the final optimization epoch.

The analysis reveals distinct behavioral differences across the three property optimization tasks. For the **immunogenicity** task, the fitness improvements are highly robust; every PROSOUNDS variant, regardless of the underlying surrogate model (MC Dropout, EDL, or FEDL) or steering configuration, achieved highly significant gains ($p < 0.005$).

The **toxicity** and **solubility** tasks represent more severe distribution shifts and stricter structural constraints, making optimization significantly harder. In the toxicity domain, while simple proportional scaling (`Prec`) struggled to achieve significance, the integration of source-domain signals via hybrid fusion (e.g., `Hybrid-Prec (EDL)`, `Hybrid-RA (FEDL)`) successfully restored statistically significant performance ($p < 0.05$). The solubility task proved to be the most challenging out-of-distribution (OOD) extrapolation. Here, only the most sophisticated uncertainty-aware strategies—specifically the hybrid proportional variants paired with MC Dropout (`Hybrid-RA (MC Dropout)`, $p = 0.0287$) and FEDL (`Hybrid-RA (FEDL)`, $p = 0.0435$)—managed to reliably and significantly outperform the baseline.

Collectively, these detailed statistical results underscore the necessity of our proposed hybrid fusion approach. Relying on target-domain steering alone is often insufficient for complex OOD tasks; however, when target signals are gated by robust epistemic uncertainty estimates (particularly via FEDL) and anchored by known source-domain representations, the PROSOUNDS framework consistently secures statistically significant advancements in protein fitness.

Supplementary Note S2: Detailed Analysis of Epistemic Uncertainty Distributions

To rigorously characterize the predictive behavior of our surrogate models and address the multi-modal nature of the uncertainty distributions, we performed a detailed Kernel Density Estimation (KDE) analysis (Figure S2). The density estimation was computed on the aggregated out-of-distribution (OOD) predictions across all three property datasets (Immunogenicity, Toxicity, and Solubility) and all three surrogate backbones (MC Dropout, EDL, and FEDL). The predictions were categorized into Correct and Incorrect outcomes, yielding a total sample size of $N \approx 4,563$. The KDE utilized a Gaussian kernel, with the bandwidth parameter estimated dynamically via Scott's rule.

Visual inspection of the resulting distributions in 2 confirms that both the Correct and Incorrect prediction outcomes exhibit multi-modal characteristics. This multi-modality is a natural reflection of the underlying data topology: the OOD sequence space is highly heterogeneous, comprising distinct local structural clusters with varying degrees of evolutionary divergence from the wild-type training manifolds. Consequently, the predictive uncertainty forms multiple density peaks across different sequence neighborhoods rather than smooth unimodal curves.

Despite this structural multi-modality, a critical macro-level trend in 3 robustly validates our uncertainty-weighted steering approach. The primary density mass of Correct predictions is firmly anchored in the low-EU regime, whereas

Table 2: Statistical significance of fitness improvements for PROSOUNDS variants compared to the baseline. A one-sided Mann-Whitney U test was performed ($N = 100$). Significant results ($p < 0.05$) are denoted in bold.

**Task**	**Surrogate Model**	**PROSOUNDS Variant**	**Mean Score**	**Baseline Score**	**U-Stat**	**p-value**	**Signif.** ($p < 0.05$)
Immunogenicity	MC Dropout	Prec	0.5344	0.4793	6083.0	**4.08e-03**	**Yes**
		RA	0.5538	0.4793	6426.0	**2.48e-04**	**Yes**
		Hybrid-Prec	0.5347	0.4793	6078.0	**4.23e-03**	**Yes**
		Hybrid-RA	0.5468	0.4793	6249.0	**1.14e-03**	**Yes**
	EDL	Prec	0.5491	0.4793	6363.0	**4.36e-04**	**Yes**
		RA	0.5483	0.4793	6260.0	**1.04e-03**	**Yes**
		Hybrid-Prec	0.5672	0.4793	6747.0	**9.89e-06**	**Yes**
		Hybrid-RA	0.5607	0.4793	6567.0	**6.47e-05**	**Yes**
	FEDL	Prec	0.5541	0.4793	6413.0	**2.79e-04**	**Yes**
		RA	0.5388	0.4793	6094.0	**3.77e-03**	**Yes**
		Hyrbid-Prec	0.5496	0.4793	6404.0	**3.03e-04**	**Yes**
		Hybrid-RA	0.5588	0.4793	6533.0	**9.04e-05**	**Yes**
Toxicity	MC Dropout	Prec	0.2229	0.2114	5721.0	**3.92e-02**	**Yes**
		RA	0.2201	0.2114	5239.0	2.80e-01	No
		Hybrid-Prec	0.2335	0.2114	5620.0	6.51e-02	No
		Hybrid-RA	0.2391	0.2114	5800.0	**2.54e-02**	**Yes**
	EDL	Prec	0.2150	0.2114	5434.0	1.45e-01	No
		RA	0.2042	0.2114	5023.0	4.78e-01	No
		Hybrid-Prec	0.2495	0.2114	5904.0	**1.36e-02**	**Yes**
		Hybrid-RA	0.2445	0.2114	5595.0	7.32e-02	No
	FEDL	Prec	0.2115	0.2114	5274.0	2.52e-01	No
		RA	0.2270	0.2114	5413.0	1.57e-01	No
		Hyrbid-Prec	0.2430	0.2114	5759.0	**3.19e-02**	**Yes**
		Hybrid-RA	0.2487	0.2114	5990.0	**7.81e-03**	**Yes**
Solubility	MC Dropout	Prec	0.2554	0.2303	5451.0	1.36e-01	No
		RA	0.2793	0.2303	5653.0	5.54e-02	No
		Hybrid-Prec	0.2668	0.2303	5472.0	1.25e-01	No
		Hybrid-RA	0.2946	0.2303	5778.0	**2.87e-02**	**Yes**
	EDL	Prec	0.2536	0.2303	5172.0	3.38e-01	No
		RA	0.2640	0.2303	5453.0	1.34e-01	No
		Hybrid-Prec	0.2228	0.2303	4649.0	8.05e-01	No
		Hybrid-RA	0.2437	0.2303	5027.0	4.74e-01	No
	FEDL	Prec	0.2444	0.2303	5324.0	2.15e-01	No
		RA	0.2409	0.2303	5273.0	2.53e-01	No
		Hybrid-Prec	0.2793	0.2303	5743.0	**3.48e-02**	**Yes**
		Hybrid-RA	0.2750	0.2303	5701.0	**4.35e-02**	**Yes**

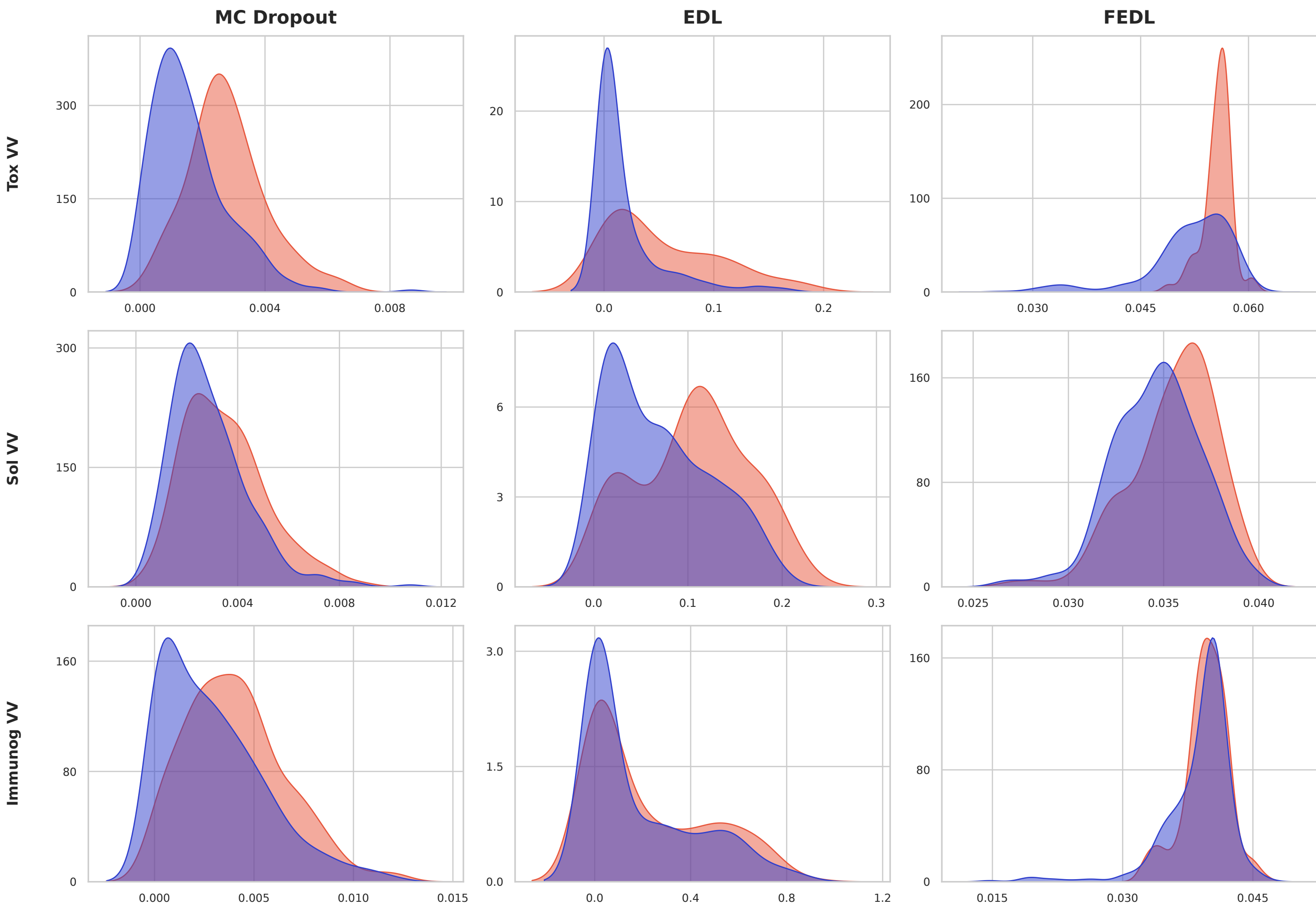

Figure 2: **Evaluation of epistemic uncertainty across diverse uncertainty-aware surrogate variants and target protein properties.** A comparison of three uncertainty estimation frameworks: MC Dropout (sampling-based), Evidential Deep Learning (EDL), and Flexible EDL (FEDL). Columns categorize the models, while rows distinguish three independent protein sequence datasets. Distributions represent estimated Epistemic Uncertainty (EU) for correct (blue) and incorrect (orange) predictions.

Incorrect predictions maintain a high density throughout the upper-EU regions. The secondary, high-EU peaks observed within the Correct distribution represent accurate predictions on extreme OOD variants where the surrogate model is, appropriately, inherently unconfident. By mathematically downweighting these high-EU regions, the PROSOUNDS framework acts as a necessary regularizer. It intentionally forfeits these highly uncertain Correct predictions to successfully shield the generative trajectory from the dense population of high-EU Incorrect predictions that would otherwise actively corrupt the functional optimization process.

References

[1] Dyah Adila, Shuai Zhang, Boran Han, and Bernie Wang. Discovering bias in latent space: An unsupervised debiasing approach. In Ruslan Salakhutdinov, Zico Kolter, Katherine Heller, Adrian Weller, Nuria Oliver, Jonathan Scarlett, and Felix Berkenkamp, editors, *Proceedings of the 41st International Conference on Machine Learning*, volume 235 of *Proceedings of Machine Learning Research*, pages 246–261. PMLR, 21–27 Jul 2024. URL `https://proceedings.mlr.press/v235/adila24a.html`.

[2] Yuanpu Cao, Tianrong Zhang, Bochuan Cao, Ziyi Yin, Lu Lin, Fenglong Ma, and Jinghui Chen. Personalized steering of large language models: Versatile steering vectors through bi-directional preference optimization. In *The Thirty-eighth Annual Conference on Neural Information Processing Systems*, 2024. URL `https://openreview.net/forum?id=7qJFkuZdYo`.

[3] Ahmed Elnaggar, Hazem Essam, Wafaa Salah-Eldin, Walid Moustafa, Mohamed Elkerdawy, Charlotte Rochereau,

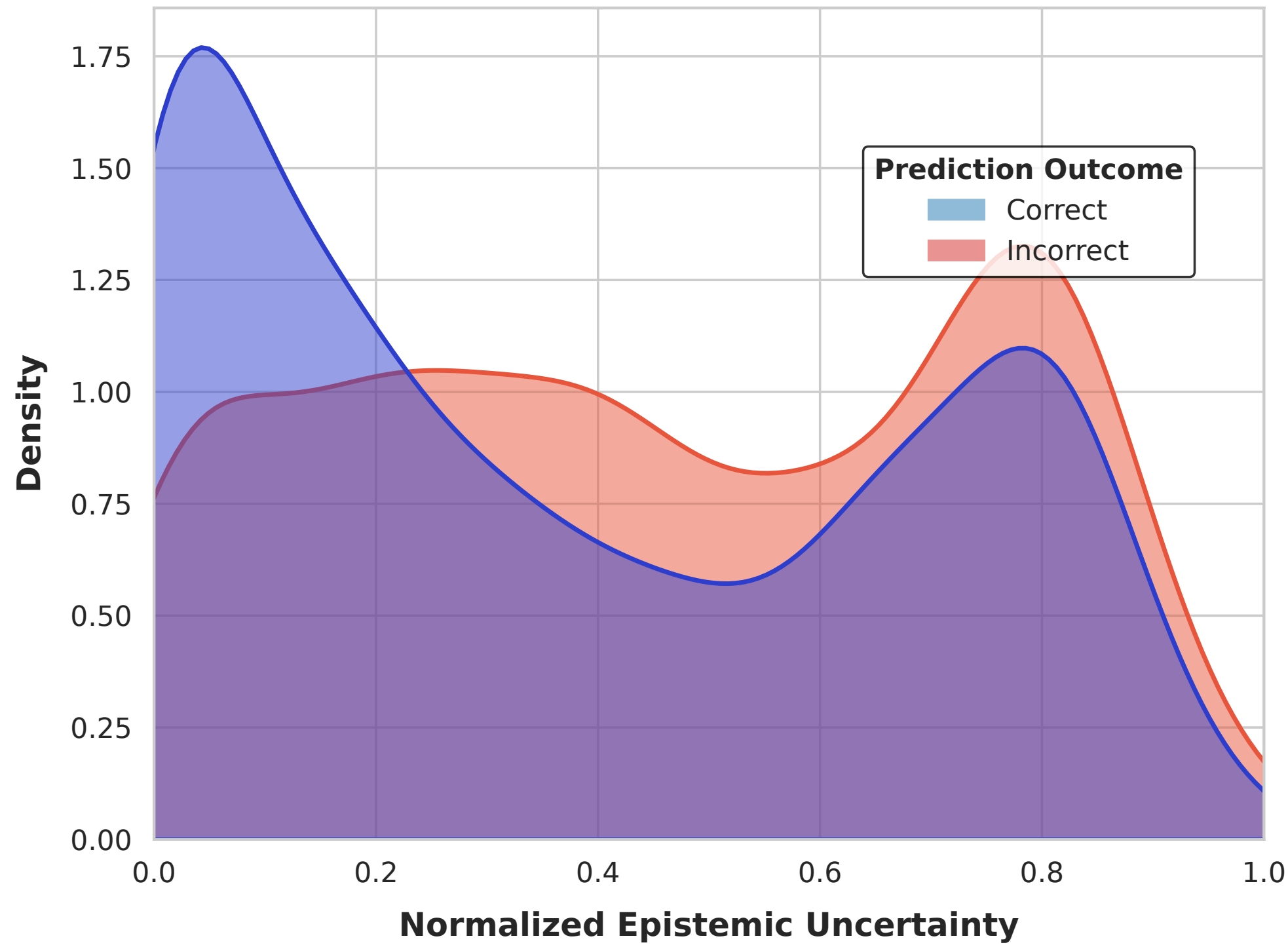

Figure 3: Density distribution of normalized epistemic uncertainty aggregated by prediction accuracy across all the property optimization tasks and all the uncertainty-aware surrogate variants. Correct predictions strongly concentrate at the lower uncertainty bounds, whereas incorrect predictions exhibit a pronounced shift toward higher uncertainty scores, demonstrating the model's ability to flag its own errors.

and Burkhard Rost. Ankh: Optimized protein language model unlocks general-purpose modelling. *arXiv preprint arXiv:2301.06568*, 2023.

[4] ESM Team. ESM Cambrian: Revealing the mysteries of proteins with unsupervised learning. `https://evolutionaryscale.ai/blog/esm-cambrian`, December 2024. EvolutionaryScale Website.

[5] Noelia Ferruz and Birte Höcker. Controllable protein design with language models. *Nature Machine Intelligence*, 4 (6):521–532, June 2022. ISSN 2522-5839. doi: 10.1038/s42256-022-00499-z. URL `http://dx.doi.org/10.1038/s42256-022-00499-z`.

[6] Noelia Ferruz, Steffen Schmidt, and Birte Höcker. Protgpt2 is a deep unsupervised language model for protein design. *Nature Communications*, 13(1), July 2022. ISSN 2041-1723. doi: 10.1038/s41467-022-32007-7. URL `http://dx.doi.org/10.1038/s41467-022-32007-7`.

[7] Thomas Hayes, Roshan Rao, Halil Akin, Nicholas J. Sofroniew, Deniz Oktay, Zeming Lin, Robert Verkuil, Vincent Q. Tran, Jonathan Deaton, Marius Wiggert, Rohil Badkundri, Irhum Shafkat, Jun Gong, Alexander Derry, Raul S. Molina, Neil Thomas, Yousuf A. Khan, Chetan Mishra, Carolyn Kim, Liam J. Bartie, Matthew Nemeth, Patrick D. Hsu, Tom Sercu, Salvatore Candido, and Alexander Rives. Simulating 500 million years of evolution with a language model. *Science*, 387(6736):850–858, 2025. doi: 10.1126/science.ads0018. URL `https://www.science.org/doi/abs/10.1126/science.ads0018`.

[8] Matthias Hein, Maksym Andriushchenko, and Julian Bitterwolf. Why relu networks yield high-confidence predictions far away from the training data and how to mitigate the problem. In *Proceedings of the IEEE/CVF conference on computer vision and pattern recognition*, pages 41–50, 2019.

[9] Brian L. Hie, Kevin K. Yang, and Peter S. Kim. Evolutionary velocity with protein language models predicts evolutionary dynamics of diverse proteins. *Cell Systems*, 13(4):274–285.e6, 2022. ISSN 2405-4712. doi: https://doi.org/10.1016/j.cels.2022.01.003. URL `https://www.sciencedirect.com/science/article/pii/S2405471222000382`.

[10] Brian L. Hie, Varun R. Shanker, Duo Xu, Theodora U. J. Bruun, Payton A. Weidenbacher, Shaogeng Tang, Wesley Wu, John E. Pak, and Peter S. Kim. Efficient evolution of human antibodies from general protein language models. *Nature Biotechnology*, 42(2):275–283, April 2023. ISSN 1546-1696. doi: 10.1038/s41587-023-01763-2. URL `http://dx.doi.org/10.1038/s41587-023-01763-2`.

[11] Long-Kai Huang, Rongyi Zhu, Bing He, and Jianhua Yao. Steering protein language models. In Aarti Singh, Maryam Fazel, Daniel Hsu, Simon Lacoste-Julien, Felix Berkenkamp, Tegan Maharaj, Kiri Wagstaff, and Jerry Zhu, editors, *Proceedings of the 42nd International Conference on Machine Learning*, volume 267 of *Proceedings of Machine Learning Research*, pages 26247–26260. PMLR, 13–19 Jul 2025. URL https://proceedings.mlr.press/v267/huang25ba.html.

[12] Rui Huang, Andrew Geng, and Yixuan Li. On the importance of gradients for detecting distributional shifts in the wild. In M. Ranzato, A. Beygelzimer, Y. Dauphin, P.S. Liang, and J. Wortman Vaughan, editors, *Advances in Neural Information Processing Systems*, volume 34, pages 677–689. Curran Associates, Inc., 2021. URL https://proceedings.neurips.cc/paper_files/paper/2021/file/063e26c670d07bb7c4d30e6fc69fe056-Paper.pdf.

[13] Agustinus Kristiadi, Matthias Hein, and Philipp Hennig. Being bayesian, even just a bit, fixes overconfidence in ReLU networks. In Hal Daumé III and Aarti Singh, editors, *Proceedings of the 37th International Conference on Machine Learning*, volume 119 of *Proceedings of Machine Learning Research*, pages 5436–5446. PMLR, 13–18 Jul 2020. URL https://proceedings.mlr.press/v119/kristiadi20a.html.

[14] Kenneth Li, Oam Patel, Fernanda Viégas, Hanspeter Pfister, and Martin Wattenberg. Inference-time intervention: Eliciting truthful answers from a language model. In *Thirty-seventh Conference on Neural Information Processing Systems*, 2023. URL https://openreview.net/forum?id=aLLuYpn83y.

[15] Song Li, Yang Tan, Song Ke, Liang Hong, and Bingxin Zhou. Immunogenicity prediction with dual attention enables vaccine target selection. In *The Thirteenth International Conference on Learning Representations*, 2025. URL https://openreview.net/forum?id=hWmwL9gizZ.

[16] Zeming Lin, Halil Akin, Roshan Rao, Brian Hie, Zhongkai Zhu, Wenting Lu, Nikita Smetanin, Robert Verkuil, Ori Kabeli, Yaniv Shmueli, Allan dos Santos Costa, Maryam Fazel-Zarandi, Tom Sercu, Salvatore Candido, and Alexander Rives. Evolutionary-scale prediction of atomic-level protein structure with a language model. *Science*, 379(6637):1123–1130, 2023. doi: 10.1126/science.ade2574. URL https://www.science.org/doi/abs/10.1126/science.ade2574.

[17] Sheng Liu, Haotian Ye, Lei Xing, and James Y. Zou. In-context vectors: Making in context learning more effective and controllable through latent space steering. In *Forty-first International Conference on Machine Learning*, 2024. URL https://openreview.net/forum?id=dJTChKgv3a.

[18] Liuzhenghao Lv, Zongying Lin, Hao Li, Yuyang Liu, Jiaxi Cui, Calvin Yu-Chian Chen, Li Yuan, and Yonghong Tian. Prollama: A protein large language model for multi-task protein language processing. *IEEE Transactions on Artificial Intelligence*, 2025.

[19] Ali Madani, Ben Krause, Eric R. Greene, Subu Subramanian, Benjamin P. Mohr, James M. Holton, Jose Luis Olmos, Caiming Xiong, Zachary Z. Sun, Richard Socher, James S. Fraser, and Nikhil Naik. Large language models generate functional protein sequences across diverse families. *Nature Biotechnology*, 41(8):1099–1106, January 2023. ISSN 1546-1696. doi: 10.1038/s41587-022-01618-2. URL http://dx.doi.org/10.1038/s41587-022-01618-2.

[20] Joshua Meier, Roshan Rao, Robert Verkuil, Jason Liu, Tom Sercu, and Alex Rives. Language models enable zero-shot prediction of the effects of mutations on protein function. In M. Ranzato, A. Beygelzimer, Y. Dauphin, P.S. Liang, and J. Wortman Vaughan, editors, *Advances in Neural Information Processing Systems*, volume 34, pages 29287–29303. Curran Associates, Inc., 2021. URL https://proceedings.neurips.cc/paper_files/paper/2021/file/f51338d736f95dd42427296047067694-Paper.pdf.

[21] Alexander Meinke and Matthias Hein. Towards neural networks that provably know when they don't know. In *International Conference on Learning Representations*, 2020. URL https://openreview.net/forum?id=ByxGkySKwH.

[22] Anh Nguyen, Jason Yosinski, and Jeff Clune. Deep neural networks are easily fooled: High confidence predictions for unrecognizable images. In *Proceedings of the IEEE conference on computer vision and pattern recognition*, pages 427–436, 2015.

[23] Nina Panickssery, Nick Gabrieli, Julian Schulz, Meg Tong, Evan Hubinger, and Alexander Matt Turner. Steering llama 2 via contrastive activation addition. *arXiv preprint arXiv:2312.06681*, 2023.

[24] Qizhi Pei, Lijun Wu, Kaiyuan Gao, Xiaozhuan Liang, Yin Fang, Jinhua Zhu, Shufang Xie, Tao Qin, and Rui Yan. BioT5+: Towards generalized biological understanding with IUPAC integration and multi-task tuning. In Lun-Wei Ku, Andre Martins, and Vivek Srikumar, editors, *Findings of the Association for Computational Linguistics: ACL 2024*, pages 1216–1240, Bangkok, Thailand, August 2024. Association for Computational Linguistics. doi: 10.18653/v1/2024.findings-acl.71. URL https://aclanthology.org/2024.findings-acl.71/.

[25] Alexander Rives, Joshua Meier, Tom Sercu, Siddharth Goyal, Zeming Lin, Jason Liu, Demi Guo, Myle Ott, C. Lawrence Zitnick, Jerry Ma, and Rob Fergus. Biological structure and function emerge from scaling unsupervised learning to 250 million protein sequences. *Proceedings of the National Academy of Sciences*, 118(15):e2016239118, 2021. doi: 10.1073/pnas.2016239118. URL https://www.pnas.org/doi/abs/10.1073/pnas.2016239118.

[26] Murat Sensoy, Lance Kaplan, and Melih Kandemir. Evidential deep learning to quantify classification uncertainty. In S. Bengio, H. Wallach, H. Larochelle, K. Grauman, N. Cesa-Bianchi, and R. Garnett, editors, *Advances in Neural Information Processing Systems*, volume 31. Curran Associates, Inc., 2018. URL https://proceedings.neurips.cc/paper_files/paper/2018/file/a981f2b708044d6fb4a71a1463242520-Paper.pdf.

[27] Shashwat Singh, Shauli Ravfogel, Jonathan Herzig, Roee Aharoni, Ryan Cotterell, and Ponnurangam Kumaraguru. Representation surgery: Theory and practice of affine steering. In *Forty-first International Conference on Machine Learning*, 2024. URL https://openreview.net/forum?id=GwA4go0Mw4.

[28] Alexander Matt Turner, Lisa Thiergart, Gavin Leech, David Udell, Juan J Vazquez, Ulisse Mini, and Monte MacDiarmid. Steering language models with activation engineering, 2025. URL https://openreview.net/forum?id=2XBPdPIcFK.

[29] Haoran Wang and Kai Shu. Trojan activation attack: Red-teaming large language models using steering vectors for safety-alignment. In *Proceedings of the 33rd ACM International Conference on Information and Knowledge Management*, CIKM '24, page 2347–2357. ACM, October 2024. doi: 10.1145/3627673.3679821. URL http://dx.doi.org/10.1145/3627673.3679821.

[30] Jingkang Yang, Kaiyang Zhou, Yixuan Li, and Ziwei Liu. Generalized out-of-distribution detection: A survey. *International Journal of Computer Vision*, 132(12):5635–5662, June 2024. ISSN 1573-1405. doi: 10.1007/s11263-024-02117-4. URL http://dx.doi.org/10.1007/s11263-024-02117-4.

[31] Jiahao Zhang, ZEQING ZHANG, Di Wang, and Lijie Hu. Controlling repetition in protein language models. In *The Fourteenth International Conference on Learning Representations*, 2026. URL https://openreview.net/forum?id=XOQxVexIJX.

[32] Andy Zou, Long Phan, Sarah Chen, James Campbell, Phillip Guo, Richard Ren, Alexander Pan, Xuwang Yin, Mantas Mazeika, Ann-Kathrin Dombrowski, et al. Representation engineering: A top-down approach to ai transparency. *arXiv preprint arXiv:2310.01405*, 2023.
